# Association of germline genetic variants in *RFC, IL15* and *VDR* genes with minimal residual disease in pediatric B-cell precursor ALL

**DOI:** 10.1038/srep29427

**Published:** 2016-07-18

**Authors:** Małgorzata Dawidowska, Maria Kosmalska, Łukasz Sędek, Aleksandra Szczepankiewicz, Magdalena Twardoch, Alicja Sonsala, Bronisława Szarzyńska-Zawadzka, Katarzyna Derwich, Monika Lejman, Katarzyna Pawelec, Agnieszka Obitko-Płudowska, Katarzyna Pawińska-Wąsikowska, Kinga Kwiecińska, Andrzej Kołtan, Agnieszka Dyla, Władysław Grzeszczak, Jerzy R. Kowalczyk, Tomasz Szczepański, Ewa Ziętkiewicz, Michał Witt

**Affiliations:** 1 Department of Molecular and Clinical Genetics, Institute of Human Genetics Polish Academy of Sciences, Poznan, Poland; 2 Department of Pediatric Hematology and Oncology, Medical University of Silesia, Zabrze, Poland; 3 Laboratory of Molecular and Cell Biology, Department of Pediatric Pulmonology, Allergy and Clinical Immunology, Poznan University of Medical Sciences, Poland; 4 Department of Pediatric Hematology, Oncology and Transplantology, University of Medical Sciences, Poznan, Poland; 5 Department of Pediatric Hematology, Oncology and Transplantology, Cytogenetic Laboratory, Children’s University Hospital, Medical University of Lublin, Poland; 6 Department of Pediatric Hematology and Oncology, Medical University of Warsaw, Poland; 7 Department of Pediatric Oncology and Hematology, Jagiellonian University Collegium Medicum, Kraków, Poland; 8 Department of Pediatric Hematology and Oncology, Collegium Medicum, Nicolaus Copernicus University in Bydgoszcz, Poland; 9 Department of Paediatric Bone Marrow Transplantation, Oncology and Hematology, Wroclaw Medical University, Wrocław, Poland; 10 Department of Internal Diseases, Diabetology and Nephrology, Medical University of Silesia, Zabrze, Poland

## Abstract

Minimal residual disease (MRD) enables reliable assessment of risk in acute lymphoblastic leukemia (ALL). However, little is known on association between MRD status and germline genetic variation. We examined 159 Caucasian (Slavic) patients with pediatric ALL, treated according to ALL-IC-BFM 2002/2009 protocols, in search for association between 23 germline polymorphisms and MRD status at day 15, day 33 and week 12, with adjustment for MRD-associated clinical covariates. Three variants were significantly associated with MRD: rs1544410 in *VDR* (MRD-day15); rs1051266 in *RFC* (MRD-day33, MRD-week12), independently and in an additive effect with rs10519613 in *IL15* (MRD-day33). The risk alleles for MRD-positivity were: A allele of *VDR* (OR = 2.37, 95%CI = 1.07–5.21, *P* = 0.03, MRD-day15); A of *RFC* (OR = 1.93, 95%CI = 1.05–3.52, *P* = 0.03, MRD-day33 and MRD-week12, *P* < 0.01); A of *IL15* (OR = 2.30, 95%CI = 1.02–5.18, *P* = 0.04, MRD-day33). The risk for MRD-day33-positive status was higher in patients with risk alleles in both *RFC* and *IL15* loci than in patients with risk alleles in one locus or no risk alleles: 2 vs. 1 (OR = 3.94, 95% CI = 1.28–12.11, *P* = 0.024), 2 vs. 0 (OR = 6.75, 95% CI = 1.61–28.39, *P* = 0.012). Germline variation in genes related to pharmacokinetics/pharmacodynamics of anti-leukemic drugs and to anti-tumor immunity of the host is associated with MRD status and might help improve risk assessment in ALL.

Acute lymphoblastic leukemia (ALL), the most common pediatric cancer accounting for approximately 30% of all childhood neoplasms, is also one of the most successfully treated malignancies in children^1^. With current treatment protocols, based on the multi-agent chemotherapy, approximately 85% of patients reach a long-term remission^2^. These relatively high cure rates have been reached through the continuous advancement of therapeutic regimens, introduction of more efficient chemotherapeutic drugs, and treatment diversification in the risk-stratified patient groups.

Quantitative assessment of the level of residual leukemic cells (minimal residual disease, MRD) in patients undergoing treatment for ALL is an important indicator of the efficiency of a therapy. Several clinical studies carried out in the late 1990s demonstrated that monitoring MRD during the first three months of treatment holds the most prognostic value for risk assessment in pediatric ALL; generally, the presence of residual leukemic cells at the specified time points is associated with inferior survival^3^,^4^,^5^. Thus, monitoring MRD allows classification of patients into risk groups, and consequently adjustment of the intensity of the therapy. Incorporation of molecular or flow cytometry-based monitoring of MRD into treatment protocols significantly improved the outcome of pediatric ALL patients^6^,^7^. Despite the discovery of novel promising prognostic factors, such as *CRLF2* expression or *IKZF1* deletions/mutations, monitoring MRD remains the most reliable indicator of the treatment efficacy in ALL^8^,^9^.

The reduction of a tumor load (which corresponds to the decreasing level of MRD) in ALL patients undergoing therapy depends both on tumor-related factors (characteristics of the leukemic cells) and on constitutive host-related factors, such as germline polymorphisms in genes involved in transport, metabolism and detoxification of chemotherapeutic agents. The vast majority of pharmacogenetics studies in ALL, searching for association of polymorphic variants with the response to the treatment, have been based on the outcome assessed through survival estimates such as EFS (event free survival) or OS (overall survival)^10^,^11^,^12^,^13^,^14^. MRD status, being assessed during the realization of a therapeutic protocol, provides a direct insight into the treatment efficacy. The use of MRD status as an indicator of the treatment response enables rapid assessment of the prognostic potential of genetic polymorphisms in association studies, with no need for a long-term follow-up that is necessary when such studies are based on survival analysis^15^.

However, very few papers have been published so far on the relation of genetic variation to MRD as a measure of the therapy outcome in ALL^16^,^17^,^18^. In the scarce published studies, MRD levels have been estimated at slightly different time points of the therapeutic regimen. In addition, most of these studies have been performed in groups of patients of diverse ethnic composition and/or treated with various protocols. Since all these factors are crucial for pharmacogenetics association studies, the results of these analyses are not fully comparable.

Here we present the findings of the search for association between the MRD status and 23 germline variants, 20 localized in genes and 3 in intergenic regions. The study was performed in a group of 159 patients diagnosed with pediatric B-cell precursor ALL (BCP-ALL) and treated according to ALL IC-BFM protocols (2002 or 2009). Importantly, the group of patients was ethnically uniform (all were Caucasian of Slavic origin), and MRD was assessed, by flow cytometry (FC) and/or real-time polymerase chain reaction (RQ-PCR), at three uniformly chosen time points after the beginning of treatment.

## Methods

### Patients

The study group consisted of 174 patients with pediatric BCP-ALL, diagnosed between June 2006 and August 2012; the median follow-up time was 3 years at the time of analyses. The criteria for inclusion into the study were: availability of a biological material (blood, bone marrow aspirates) for genotyping of polymorphic loci, and availability of MRD data at day 15 (early assessment of the response to the remission induction), day 33 (assessment at the end of the remission induction), and week 12 (assessment before the beginning of the consolidation treatment). It has to be noted that, for the majority of Polish patients admitted to the clinics between 2006 and 2012, assessment of MRD was not obligatory during ALL treatment procedure. Since the availability of MRD data was a key inclusion criterion in our study, the study group did not consist of the consecutive patients.

The patients were treated according to ALL IC-BFM 2002 protocol (146 patients; 83.9%) or according to ALL IC-BFM 2009 protocol (28 patients; 16.1%)^19^. In the 2002 protocol, classification of patients into the risk group was based solely on clinical and genetic features identified at diagnosis and during the early phase of treatment (remission induction). In the 2009 protocol, these prognostic features were retained, but the flow cytometry (FC)-based assessment of MRD at day 15 was added as an obligatory element of the procedure, and used as one of the criteria in the risk group classification performed after day 33.

With the treatment scheme for the remission induction remaining unchanged until day 33, the data on MRD at day 15 and at day 33 from patients treated according to either of the two protocols could be included in the association analysis. On the other hand, six patients out of the 28 treated according to the 2009 protocol had to be excluded from the analysis of association with the MRD status at week 12. These were patients classified into the high risk group and thus, unlike in the 2002 protocol, receiving augmented protocol IB after day 33 (in Poland the augmented protocol is only administrated to patients in the high risk group).

The study was approved by the Ethics Committee of Poznan University of Medical Sciences (resolution number 1013/09). All experiments were conducted in accordance with the relevant guidelines and regulations, approved by this Committee. All patients or their guardians expressed an informed consent for participation in the study.

### MRD assessment

MRD data obtained by standardized quantitative methods was available for 159 of 174 patients (91.4%), and was based on flow cytometry (further referred to as MRD_FC_) in 136 patients (78.1%) and on real-time PCR detection of Ig/TCR gene rearrangements (further referred to as MRD_RQ_) in 23 patients (13.2%). For 15 patients (8.6%), the only available MRD data was obtained by a simplified, low-cost method using PCR-based detection of Ig/TCR gene rearrangements and clonality assessment by heteroduplex analysis^20^; these patients were excluded from the association analyses.

For MRD data obtained by either FC or RQ, a common cut-off level of 10^−4^ (1 malignant cell/10 000 normal cells) was used to define patients with MRD-positive and MRD-negative status (MRD > = 10^−4^ and MRD < 10^−4^, respectively). This cut-off level, often regarded as a sensitivity threshold of the FC method, has been earlier used in other studies on the association of polymorphic variants with MRD measured by FC and/or RQ^16^,^17^,^18^.

Assessment of MRD_FC_ was performed in full bone marrow aspirates, according to the standard protocols of the EuroFlow Group, using a single or double 8-color combination of standardized monoclonal antibodies and 8-colour Becton Dickinson FACS Canto II flow cytometer^21^,^22^. For molecular analyses, mononuclear cells were isolated from the bone marrow using density gradient centrifugation. Real-time PCR analysis of MRD was carried out according to standard EuroMRD-ALL protocols and data interpretation guidelines^23^, with the use of standardized BIOMED-1 and BIOMED-2 primers^24^,^25^ for *IGH*, *IGK*-Kde, *TCRG*, *TCRD* and *TCRB* loci (the last with the application of TCRB Gene Clonality Assay-Gel Detection; Invivoscribe). 7900HT Fast Real-Time PCR System (Life Technologies) was used for the quantification of the MRD levels.

### Genotyping of polymorphic loci

Selection of 23 polymorphic markers for the association study was based on the combination of two approaches. In a candidate gene approach, the genes were selected based on their involvement in metabolic pathways of drugs used in ALL therapy, as evidenced by Pharmacogenomics Knowledgebase (www.pharmgkb.org) and literature data. The polymorphisms included: *GSTM1* and *GSTT1* gene deletions, *GSTP1* (rs1695), *MTHFR* (rs1801133), *TYMS* 28-bp tandem repeats (rs3474033: allele TSER*2, allele TSER*3, and a G > C SNP within the TSER*3 allele; TSER*3G > C), *RFC* (rs1051266), *NR3C1* (rs6198, rs41423247), *MDR1* (rs3789243, rs2235046, rs1045642), and *VDR* (rs2228570, rs1544410). The second approach allowed inclusion of polymorphisms in the genes earlier reported to be associated with the MRD-positive status: *CCR5* (rs333 also known as 585del32)^16^*, TPMT* (rs1800460, rs1142345)^17^; other six loci from the genome-wide association study (GWAS)^18^: *IL15* (rs10519613), *NALCN* (rs7992226), *CCDC85C* (rs11160533), and three intergenic (rs3862227, rs4888024, rs9871556). The six loci selected from the GWAS study^18^ were chosen with regard to their *P*-values for association with MRD, odds ratio (OR) for the MRD-positive status associated with carrying the risk allele, *P*-values for association with other phenotypes (e.g. methotrexate clearance), and minor allele frequency (MAF) in Caucasians.

Genotyping of polymorphic loci was performed in all 174 patients. DNA was isolated with the use of QIAamp DNA Blood Mini Kit (Qiagen, Germany) from blood/bone marrow samples obtained at the time of hematological remission (in most cases, week 12). High Resolution Melting (HRM) analysis was used for genotyping of 11 loci. Six loci were studied with the use of TaqMan Genotyping Assays (Life Technologies), namely: *NALCN, CCDC85C*, three intergenic variants selected from GWAS and one polymorphism in *NR3C1* (rs41423247). 7900HT Fast Real-Time PCR System was used for both HRM and TaqMan Genotyping assays. Conventional PCR and agarose gel detection was used for genotyping of *CCR5* (585del32), *GSTM1* and *GSTT1* gene deletions, *TYMS* 28-bp tandem repeats (alleles TSER*2, TSER*3, TSER*3G > C) and *RFC*, the latter two followed by restriction fragments length polymorphism analysis. For each polymorphic locus, 10% of results were verified by repeating the genotyping analysis or by direct sequencing.

All polymorphic loci were screened for linkage disequilibrium (LD) using SNAP software (SNP Annotation and Proxy Search, Broad Institute) based on Haploview 4.0 and genotype data from International HapMap Project and the 1000 Genomes Project^26^. Compliance of genotypes distribution with the Hardy-Weinberg equilibrium (not applicable to *GSTM1* and *GSTT1* gene deletions) was tested using the Court Lab Calculator (http://www.tufts.edu/). PolyPhen-2 (http://genetics.bwh.harvard.edu/pph2/) and SIFT (http://sift.jcvi.org/) were used for prediction of functional effects of selected polymorphic variants.

### Statistical analyses

MRD results were analyzed as a categorical variable (MRD-positive vs. MRD-negative, with a cut off level 10^−4^) and as a continuous variable (MRD levels).

Pearson χ^2^ test or, if expected frequencies were <5, 2-sided Fisher’s exact probability test (2 × 2 tables) and Freeman-Halton extension of 2-sided Fisher’s exact probability test (2 × 3 tables), with 2-sided Z-test for difference in proportions as a *post hoc* test were used to estimate the significance of: 1/differences in genotypes distribution between patients with MRD-positive and MRD-negative status; 2/association of MRD status with the allele frequency and allele positivity (genotypes pooled into binary groups and analyzed in dominant and recessive mode, e.g. GG + AG < > AA and AA + AG < > GG); odds ratio (OR) and confidence intervals (CI) for MRD-positivity calculated for the allele frequency and allele positivity; 3/association of the clinical features with MRD status, defined as ‘MRD-associated clinical covariates’; 4/additive effects between polymorphic variants (differences between MRD-positive and MRD-negative patient groups in the distribution of patients carrying risk allele in 0, 1 or 2 loci identified to be associated with the MRD status at day 33).

Univariate logistic regression was used to confirm the association of polymorphic variants with MRD status. Multivariate logistic regression was used to adjust for MRD-associated clinical covariates and for identification of the best regression models predicting the MRD status.

Non-parametric test (Kruskal-Wallis ANOVA on ranks) was used for analysis of association of polymorphic variants (genotypes and binary groups representing allele positivity) with MRD levels (MRD as a continuous variable).

Two-sided analysis of statistical power was performed using Quanto software v.1.2.4.

*P*-values < 0.05 defined statistical significance in all tests applied.

Benjamini-Hochberg procedure was applied to control for false discovery rate (FDR) in case of multiple comparison tests (association of the MRD status with the distribution of genotypes, allele frequency and allele positivity)^27^,^28^. This adjustment was not applied to tests involving clinical covariates and additive effects of carrying risk alleles, since they did not comply with the Benjamini-Hochberg assumption of the independence of multiple tests.

Statistica v. 10 (StatSoft Inc.) and *VassarStats*: *Website for Statistical Computation* (http://vassarstats.net/) were used for statistical analyses.

## Results

### MRD-associated clinical covariates

There are several clinical covariates of prognostic significance that have been widely used for risk group classification in ALL (e.g. in the ALL IC-BFM 2002 protocol). Many of them have been retained in the recent protocols that include MRD monitoring (such as ALL IC-BFM 2009). They include parameters identified at diagnosis, such as: age, white blood cell count (WBC), the presence of translocations t(9;22)/*BCR-ABL*, t(4;11)/*MLL-AF*, or both, (further referred to as ‘fusion genes’), and factors assessed early during therapy, such as ALL blast count after 8 days of prednisone treatment (prednisone response) and bone marrow morphology (M1, M2, M3) assessed at day 15 and day 33.

Distribution of our patients according to the clinical features at diagnosis and early induction, presented as prognostic categories, is shown in Table 1. Although the study group did not consist of consecutive patients (see *Methods*, section *Patients*), their distribution into high risk (HR), intermediate (IR) and standard risk (SR) groups (Table 1) did not differ from that reported for a cohort of 5060 patients treated according to the ALL IC-BFM 2002 protocol^19^ (*P* = 0.2265).

The distribution of patients according to the MRD status assessed at day 15, day 33 and week 12 following the beginning of the treatment, measured by FC (MRD_FC_) and – in an enlarged group of patients – by FC or RQ (MRD_FC/RQ_), is presented in Table 2.

The analysis of association between the clinical covariates and the MRD status (MRD_FC_ and MRD_FC/RQ_) was estimated at three time points (Table 3).

The risk group (according to ALL IC-BFM criteria, Table 1) was the only covariate associated with MRD_FC/RQ_ positivity at day 15 (*P* = 0.044); there was no association (*P* = 0.067) with MRD_FC_.

The risk group was also significantly associated with MRD_FC_ at day 33 and at week 12 (*P* < 0.001, *P* < 0.001, respectively). Significant associations with MRD_FC_ at these time points were also found for the status of the ‘fusion genes’ (*P* = 0.001, *P* = 0.021, respectively) and bone marrow morphology at day 15 (*P* = 0.002, *P* = 0.001, respectively). The same clinical covariates were found significantly associated with MRD at these time points in the enlarged group of patients (MRD_FC/RQ_), with one additional covariate (prednisone response) at day 33.

All these covariates were further used in multivariate analysis (logistic regression) with polymorphic loci associated with MRD status. Risk group was assessed separately in logistic regression model, since it is related to the presence of fusion genes and bone marrow morphology at day 15.

### Identification of MRD-associated polymorphic loci

All but two polymorphic loci from the 23 analyzed in our study were in Hardy-Weinberg (HW) equilibrium. The rs1142345 in *TPMT* gene deviated from the HW equilibrium, but was not associated with the MRD status in our study group. The rs1800460 in the same gene, found non-polymorphic in our cohort, was excluded from the analyses. No linkage disequilibrium (LD) between any of the polymorphic loci was observed.

The association of all polymorphic loci with MRD_FC_ was initially tested by analyzing the distribution of genotypes among patients with MRD-positive and MRD-negative status. Two loci were significantly associated with the MRD_FC_ status: rs10519613 in *IL15* with MRD at day 33 (*P* = 0.029) and rs1051266 in *RFC* with MRD at week 12 (*P* = 0.004). The 2-sided Z-test for difference in proportions revealed that CC and AC genotypes of the *IL15* locus were significantly more frequent among patients with the negative MRD status at day 33 (*P* = 0.019) and the positive MRD status at day 33 (*P* = 0.013), respectively; while there was no significant difference in the distribution of AA genotype (*P* = 0.079). AA genotype of the *RFC* locus was significantly more frequent among patients with the positive MRD status at week 12 (*P* = 0.001), while there was no significant difference in the distribution of AG and GG genotypes (*P* = 0.082 and *P* = 0.170, respectively). A marginally significant associations (*P* values < 0.08) were also observed for rs1544410 in *VDR* with MRD at day 15 and at week 12 (*P* = 0.078 and *P* = 0.064, respectively) (Table 4). All the associated loci are further often referred to by the gene names only, with the omission of ‘rs’ identifiers.

The above associations of genotypes with the MRD_FC_ status were subsequently analyzed in univariate logistic regression, and were confirmed for: *VDR* and MRD at day 15 (*P* = 0.034), *IL15* and MRD at day 33 (*P* = 0.039) and *RFC* and MRD at day 33 (*P* = 0.047). A significant association with MRD was also confirmed in the enlarged group of patients (MRD_FC/RQ_) for: *VDR* and MRD at day15 (*P* = 0.026), *RFC* and MRD at day 33 and at week 12 (*P* *=* 0.048 and *P* *=* 0.037, respectively).

The association of all the loci under study with MRD_FC_ status was subsequently investigated by the analysis of differences in allele frequencies (not applicable to *GSTM1* and *GSTT1* gene deletions) (Table 5). Significant difference in the allele frequency in MRD-positive and MRD-negative patients was found for: *VDR* (allele A and positive MRD status at day 15; OR = 2.37, 95% CI = 1.07–5.21, *P* = 0.03), *RFC* (allele A and positive MRD status at day 33; OR = 1.93, 95% CI = 1.05–3.52, *P* = 0.03) and *IL15* (allele A and positive MRD status at day 33; OR = 2.30, 95% CI = 1.02–5.18, *P* = 0.04). These results were confirmed by 2-sided Z-test for difference in proportions: for the association of allele frequencies in *VDR* with MRD at day 15, *IL15* with MRD at day 33 and *RFC* with MRD at day 33 (*P* = 0.028, *P* = 0.039, *P* = 0.030, respectively).

Significant difference was also found for *RFC* allele frequencies in patients with MRD-positive and MRD-negative status at week 12 (*P* < 0.01). Although it was not possible to calculate OR due to 0 value in one of the cells, the association of A allele with positive MRD status at week 12 was confirmed with 2-sided Z-test for difference in proportions: *P* = 0.003.

Statistical power for these associations ranged from 70% for the *VDR* locus (MRD at day 15) to 57% and 56% for *RFC* and *IL15* loci (MRD at day 33). It was not possible to calculate statistical power for *RFC* locus associated with MRD at week 12 (OR was non evaluable).

The associations of *VDR*, *IL15* and *RFC* alleles with the MRD_FC_ status were further investigated by the analysis of differences in allele positivity (genotypes represented as binary groups) in patients with the MRD-positive and MRD-negative status (Table 6). A significant association was found for *IL15* (positivity for the minor allele A with the positive MRD status at day 33, as revealed by AA + AC < > CC analysis, OR = 2.91, 95% CI = 1.17–7.27, *P* = 0.024, confirming A as the risk allele) and *RFC* (positivity for the major allele G associated with the negative MRD status at day 33; GG + AG < > AA, OR = 0.37, 95% CI = 0.15–0.92, *P* = 0.043, indicating G as a protective allele. Positivity for the G allele was also associated with the negative MRD status at week 12, *P* = 0.004, with no possibility to calculate OR due to 0 value in one of the cells). These results were confirmed by 2-sided Z-test for difference in proportions: for *IL15* locus (association with the MRD status at day 33, *P* = 0.019) and *RFC* locus (association with the MRD status at day 33 and week 12, *P* = 0.029 and *P* = 0.001, respectively).

The *VDR*, *IL15*, and *RFC* loci were also analyzed for association with the MRD regarded as a continuous variable (MRD levels tested with the use of Kruskal-Wallis ANOVA on ranks). The following associations with MRD_FC_ were found: *IL15* with the MRD status at day 33 (*P* = 0.037 for genotypes distribution, and *P* = 0.013 for AA + AC < > CC); *RFC* with the MRD status at day 33 (*P* = 0.049 for GG + GA < > AA), and *RFC* with the MRD status at week 12 (*P* = 0.003 for genotypes distribution; *P* = 0.001 for GG + GA < > AA). The associations of *RFC* with the MRD status at week 12 were also confirmed in the enlarged group of patients (MRD_FC/RQ_; *P* = 0.030 for genotypes distribution and *P* = 0.009 for GG + GA < > AA). These results were consistent with our findings on the association of these variants with MRD as a categorical variable (MRD-positive vs. MRD-negative status). No statistically significant association was found between MRD levels and *VDR* locus.

### Adjustment for MRD-associated clinical covariates

MRD-associated loci (genotypes and/or binary groups) were then analyzed in multivariate analysis to adjust for clinical covariates significantly associated with the MRD status in our cohort of patients (Table 3), and to assess if the inclusion of these loci improves prediction of the MRD status in logistic regression models.

Association of *RFC* genotypes and binary groups with the MRD status at day 33 remained significant after adjustment for the clinically assessed risk group (ALL IC-BFM 2002/2009 risk group). Inclusion of data on the distribution of *RFC* genotypes or binary groups improved logistic regression models for the MRD status prediction (in the group of patients with the MRD_FC_ data: *P* < 0.00001 for the models combining *RFC* data with the risk group vs. *P* = 0.00001 for the model including risk group alone). However, in the larger cohort of patients (MRD_FC/RQ_ data), the model including risk group alone was the best model predicting the MRD status. After adjustment for the fusion genes and the bone marrow morphology at day 15, *RFC* locus had no significance in association with the MRD status at day 33.

Association of *RFC* genotypes with the MRD status at week 12 remained significant after adjustment for the fusion genes combined with the bone marrow morphology at day 15, as well as after adjustment for the risk group. Inclusion of *RFC* genotypes data resulted in the improvement of the logistic regression model (in the group of patients with the MRD_FC/RQ_ data, *P* < 0.00001 vs. *P* = 0.00001 for the model with fusion genes combined with bone marrow morphology alone; *P* < 0.00001 also for the model with the risk group alone). Distribution of the binary groups (GG + AG < > AA) in *RFC* locus remained significantly associated with the MRD_FC_ status at week 12 after adjustment for the clinical covariates: fusion genes and bone marrow morphology (*P* = 0.0078) or the risk group (*P* = 0.0012). However, inclusion of *RFC* binary groups did not improve the logistic regression models as compared to those with clinical covariates only (*P* = 0.0001 for the model including fusion genes and bone marrow morphology alone, and *P* = 0.00004 for the model including risk group alone). In the enlarged group of patients with MRD_FC/RQ_ results, the association of GG + AG < > AA distribution in *RFC* locus remained significant only after adjustment for the risk group (*P* = 0.03848) but did not improve the logistic regression model including risk group alone (*P* < 0.00001).

The distribution of *VDR* genotypes remained significantly associated with the MRD_FC/RQ_ status at day 15 after the adjustment for the risk group (*P* = 0.01720, for logistic regression model).

Association of genotypes and binary groups in *IL15* locus with the MRD status at day 33 did not remain significant (neither for MRD_FC_ nor MRD_FC/RQ_) after adjustment for the clinical covariates.

### Additive effect of *IL15* and *RFC* regarding MRD status at day 33

*IL15* and *RFC* loci, with the identified risk alleles associated with the positive MRD_FC_ status at day 33, were analyzed for a possible additive effect (Table 7). A significant difference in the distribution of patients carrying genotypes with risk alleles in both these loci, in only one or in none of these two loci (further referred to as 2, 1 and 0 risk allele-carrying genotypes, respectively) was found between patients with MRD-positive and MRD-negative status (*P* = 0.017). A significant difference was also found in the distribution of patients with 2 vs. 1 and 2 vs. 0 genotypes among groups with positive and negative MRD_FC_. Odds ratio for the positive MRD status was: OR = 3.94 (95% CI = 1.28–12.11, *P* = 0.024) for patients with 2 risk allele-carrying genotypes compared to those with 1 risk allele-carrying genotype, and OR = 6.75 (95% CI = 1.61–28.39, *P* = 0.012) for patients with 2 risk allele-carrying genotype compared to patients carrying no risk allele. These findings were confirmed in the enlarged group of patients (association with the positive MRD_FC/RQ_ status): the significant difference in the distribution of patients with 2 vs. 1 vs. 0 risk allele-carrying genotypes among MRD-positive and MRD-negative groups (*P* = 0.031); OR = 2.90 (95% CI = 1.09–7.68, *P* = 0.038) for 2 vs. 1; OR = 4.56 (95% CI = 1.39–14.97, *P* = 0.017) for 2 vs. 0. This additive effect of *RFC* and *IL15* remained significant in multivariate analysis (logistic regression) after adjustment for the MRD-associated clinical covariates: fusion genes and bone marrow morphology at day 15 (*P* = 0.00002 and *P* < 0.00001 for models analyzing association with MRD_FC_ and MRD_FC/RQ_, respectively) and after adjustment for the risk group (*P* < 0.00001 for logistic regression model analyzing association with MRD_FC_).

## Discussion

Most of the data on the association of germline genetic variation in ALL patients with the treatment outcome comes from the research focused on the genes involved in metabolic pathways of drugs used in ALL therapy (a candidate gene approach), with standard indicators (survival analysis) used as a measure of therapy efficacy^10^,^11^,^12^,^13^,^14^.

Despite the fact that MRD status is a direct and rapid indicator of treatment results, the association of polymorphic variants with MRD has not been extensively investigated, with only three studies published so far^16^,^17^,^18^. The existing scarce data on the MRD-associated genetic variants have been obtained in groups of patients of diverse ethnic composition and treated with various therapeutic protocols, therefore with different time points of MRD assessment used. These factors might substantially influence the results of a pharmacogenetics analysis.

In contrast, our study was performed in the ethnically homogeneous patient group (Caucasians of Slavic origin), using uniform time points of MRD assessment and the cut-off level of 10^−4^ . Comparison of our study design with other studies^16^,^17^,^18^ focused on MRD-associated germline polymorphisms is presented in Table 8.

We searched for the association of the germline genetic variants in selected genomic regions with the MRD status, used as a direct measure of the ALL therapy outcome. The cohort comprised 159 patients, diagnosed with pediatric BCP-ALL and treated according to ALL IC-BFM 2002 and ALL IC-BFM 2009 protocols. The regimen used in both protocols enables direct inclusion into the association analysis of MRD results at two of the three time points assessed in our study; for the analysis of the third time point, several patients treated with the ALL IC-BFM 2009 protocol were excluded. The time points used in the present study were: early assessment of the response to initial treatment (remission induction, day 15), the end of remission induction (day 33), and the time point before introduction of the consolidation treatment (week 12). MRD was assessed by FC (in the majority of patients) and by RQ-PCR (to extend the number of patients with available MRD results); in both groups the common cut-off level was10^−4^.

We demonstrated the association of the MRD status with the genetic variants in three genes: *VDR*, *RFC* and *IL15*. The rs1544410 variant in *VDR* was significantly associated with the MRD status at day 15. *VDR* (vitamin D receptor) locus encodes a steroid hormone receptor, which upon binding an active metabolite of vitamin D (1 alpha, 25-dihydroxyvitamin D-3) exerts a pleiotropic effect on a wide range of biological processes, including calcium homeostasis but also modulation of immune response, cell differentiation and proliferation^29^. VDR, in complex with the active form of vitamin D, acts as a *trans*-acting transcriptional regulatory factor through binding to ‘vitamin D response elements’ (VDRE). VDRE are present in promoter regions of over 150 human target genes^30^, including *CYP3A4*, which metabolizes a number of therapeutic agents used in ALL treatment^31^. Due to the role of vitamin D in the immune system homeostatis, the genetic variation in *VDR* has also been studied in association with the outcome of autoimmune diseases^32^.

Two *VDR* gene variants analyzed in the present study (rs1544410 also named *Bsm*I, and rs2228570 named *Fok*I, both localized in the 3′ end of *VDR* gene, though in different LD blocks) are among *VDR* variants broadly investigated for association with the risk and outcome of several malignancies, including glioma^33^, colon cancer^34^ and ALL^13^. In the latter study, T allele of rs2228570 and GG genotype of rs1544410 have been associated with the increased risk of central nervous system relapse in pediatric ALL patients in the higher-risk arm^13^. This contradicts our study results based on the allele frequency analysis in MRD-positive vs. MRD-negative groups, whereby no significant association of rs2228570 was found and the opposite A allele of rs1544410 was identified as a risk allele. The possibility that these discrepant findings are due to the ethnic differences between the groups studied (a non-uniform group of patients with Caucasian, African American and other ethnicities studied by Rocha *et al*.^13^ and the homogeneous group of Slavic patients studied by us) could be related to the fact that the composition of LD blocks in the *VDR* gene is different in Caucasians, African Americans and Asians. However, in all these populations the rs2228570 variant is not assigned to any of the LD blocks, while the rs1544410 variant resides in the distal 3′ haplotype block^35^. The discrepant results regarding the risk alleles in *VDR* variants might be due to the different phenotypes analyzed: MRD positive status in the bone marrow in our study and central nervous system relapse in the study by Rocha *et al*.^13^. In fact, although no functional effect of the rs1544410 variant is known so far, it has been postulated that association with various phenotypes might reflect linkage disequilibrium of this marker variant with a causative variant in or near the *VDR* gene^36^. The pleiotropic regulatory function of *VDR* gene might explain why the same allele is identified as a risk allele for one phenotype, while being a protective allele for another.

Two other variants, rs10519613 in *IL15* and rs1051266 in *RFC*, were found to be significantly associated in our cohort with the MRD status at day 33 (and at week 12, for *RFC*).

The association of *RFC* with MRD was very consistent throughout all the analyses. It was found both in the smaller group of patients assessed by FC only and in the enlarged group assesses by FC/RQ. It was shown both for MRD considered as a categorical variable (positive vs. negative) and as a continuous variable, and for *RFC* genotypes distribution, allele frequency and allele positivity. In multivariate analysis, *RFC* remained significantly associated with the MRD_FC_ and MRD_FC/RQ_ status at day 33 after adjustment for the risk group; also the association of the *RFC* genotypes and binary groups with MRD at week 12 remained significant after adjustment for MRD-associated clinical covariates. The inclusion of *RFC* data improved the logistic regression model for the MRD status at day 33 and at week 12, as compared to the models containing clinical covariates alone.

*RFC* gene (reduced folate carrier, also named *SLC19A1* for solute carrier family 19 member 1), encodes the membrane protein responsible for carrier-mediated transport of folate, which is essential for DNA synthesis, DNA repair and DNA methylation. Methotrexate (MTX), a key anti-metabolite component of chemotherapeutic protocols used in ALL, is also actively transported by RFC protein.

The rs1051266 variant (80G > A; Arg27His in exon 2 of *RFC*) has been widely studied for correlation with MTX efficacy and toxicity in various diseases, including rheumatoid arthritis, colorectal neoplasms and ALL. Some studies investigated its association with ALL susceptibility^37^ and outcome^38^,^39^, with contradictory results. The A allele of rs1051266 has been shown to be associated with better response to MTX treatment and improved outcome in rheumatoid arthritis^40^ and in ALL^38^,^39^,^41^. None of these ALL studies used MRD as an indicator of the ALL outcome. In a single study, rs1051266 has been studied in relation to MRD levels in children with ALL treated according to Pediatric Oncology Group protocols, but no association was found^16^.

In contrast to these findings, we demonstrated that A allele of rs1051266 was associated with the MRD positive status at day 33 and week 12, indicating that it is a risk allele for the inferior response to treatment in ALL. Our results are in line with those of two other groups, who did not use MRD as an indicator of ALL outcome, but have indicated A allele of rs1051266 as a risk allele for the pediatric ALL outcome^42^,^43^. One has demonstrated poorer survival and significantly increased chance for ALL relapse in patients with GA and AA genotype as compared to patients with GG genotype^42^. In the other study, significantly higher risk of event in EFS analysis has been shown for patients carrying A allele as compared to GG genotype; this remained significant after adjustment for other prognostic factors that influence ALL outcome^43^. The authors have also shown higher MTX plasma levels in patients with AA genotype than in patients with other genotypes, and speculated that the impaired intracellular uptake might be due to the lower affinity of the resulting variant of RFC protein to MTX. However, *in vitro* study has shown no significant differences in MTX uptake between transport-impaired K562 cells transfected with either 80GG or 80AA *RFC* cDNA^44^. The latter observation is in line with our *in silico* prediction of a functional effect of rs1051266 in RFC, which was ‘benign’ according to PolyPhen-2 (http://genetics.bwh.harvard.edu/pph2/) and ‘tolerated’ according to SIFT algorithms (http://sift.jcvi.org/).

The rs10519613 variant in *IL15*, has been previously shown to be associated with MRD levels in pediatric ALL. In GWAS analysis, performed in pediatric ALL patients treated according to St Jude Total Therapy and Children’s Oncology Group protocols, rs10519613 (together with five other SNPs in *IL15*, all in strong LD, r^2^ > 0.5) was among the top 102 variants associated with the MRD levels at the end of induction^18^. The A allele of rs10519613 has been identified as a risk allele for MRD-positive status, which is in line with our findings. Recently, *IL15* locus has been demonstrated to be associated with EFS in pediatric patients treated according to Malaysia-Singapore ALL 2003 protocol (a modification of ALL IC-BFM 2002 protocol)^10^. The rs17015014 variant of *IL15* reported to be associated with ALL outcome^10^, was also one of the five linked SNPs previously reported to be MRD-associated^18^. Although the study design differed from ours, being focused on monitoring survival rather than MRD as the end point for the studied associations, our results remain in line with these findings. Thus, we provide additional data supporting the role of *IL15* polymorphisms in the treatment response in pediatric ALL.

*IL15*, interleukin 15, encodes the cytokine that regulates activation and proliferation of T lymphocytes and natural killer (NK) cells, and thus is involved in immune response, including anti-tumor response^45^,^46^. Natural killer cells are key players in the innate immune response against cancer and virus-infected cells^47^,^48^. There has been recently an increasing number of data on the use of genetically manipulated NK cells, including NK cells expressing membrane-bound IL15 for autocrine stimulation. This results in better persistence and expansion of NK cells following infusion into the patient and improves their anti-tumor potential in adoptive immunotherapy^48^,^49^,^50^,^51^. These very latest functional experiments are an elegant evidence for the role of *IL15* gene in anti-tumor response, and are in line with our results on the association of *IL15* polymorphism with MRD-status.

An important finding of our study concerns the additive effect of risk alleles in *RFC* locus (allele A of rs1051266) and *IL15* (allele A of rs10519613) for the association with the positive MRD_FC_ status at day 33 (the end of remission induction in ALL IC-BFM protocols 2002 and 2009). Patients carrying at least two risk alleles in these two loci had a significantly higher (nearly 4-fold difference) chance for the positive MRD status as compared to patients with risk allele/s in any of these two genes and to those with no risk alleles (over 6-fold difference). This additive effect was confirmed in the enlarged cohort of patients with MRD_FC/RQ_ results and remained significant after the adjustment for MRD-associated clinical covariates in multivariate analysis.

There are some limitations of the current study that should be noted. The main limitation is a relatively small sample size, related to the inclusion criteria we used: Caucasian patients of Slavic origin, treated according to ALL IC BFM protocols, with MRD data available for three time points during the treatment. Due to the limited sample size, the statistical power of the tests for associations ranged from 5% to 99% (Table 5). The power of tests regarding *VDR*, *RFC* and *IL15* loci (found to be associated with the MRD status) ranged from 56–70%, but was still lower than the 80%, usually appreciated in association studies. As a consequence, the study could only offer a limited possibility to detect MRD association with small-effect polymorphisms (OR: 1.1–1.5; this translated to the increased risk of false negatives) and low positive predictive value (PPV, the probability that an observed effect is a true positive).

However, as in any association study, the consequences of the low power should be weighted in light of the study goals^52^,^53^. Here we aimed to identify candidate polymorphic variants that might serve as potential prognostic markers of the MRD status. Confronted with the rarity of patients satisfying the inclusion criteria on one side and the substantial impact that could be potentially gained from the identification of novel genetic predictors of the therapy efficacy, we reported our findings in spite of being aware of the power limitations.

In particular, the association with the MRD status was reported for three loci: *VDR*, *IL15* and *RFC*, of which only one (*RFC*) remained significant upon Benjamini-Hochberg adjustment for multiple testing. The advantage of reporting marginally significant results was substantiated by our finding of the additive effect of carrying risk alleles in *IL15* and *RFC* loci. This indicated that while a single polymorphic locus may add little to the prognostic significance of the existing prognostic factors, concurrent analysis of several MRD-associated loci might improve risk assessment in ALL patients. Obviously, our findings require confirmation in larger cohorts of patients, taking into account different ethnicities and treatment protocols.

Another limitation of our study is associated with the use of arbitrary cut-off level of 10^−4^, which was chosen to categorize MRD results (MRD-positivity vs. MRD-negativity) and to compare with the results of others, but which does not fully reflect MRD-based classification into three risk groups, used in the clinical practice.

## Conclusions

Our results indicate associations of polymorphic variants in three genes with the MRD-status in pediatric ALL patents: rs1544410 in *VDR* with MRD at day 15; rs1051266 in *RFC*, independently and in combination with rs10519613 in *IL15* with MRD at day 33; and rs1051266 in *RFC* with MRD at week 12. The association of the *RFC* variant was the most consistent in our study, and remained significant also upon the adjustment for multiple testing. We showed an additive effect of the risk alleles in *RFC* and *IL15* on the strength of their association with MRD at day 33. We also identified clinical features associated with the MRD status in ALL IC-BFM 2002 and 2009 protocols.

Although association studies do not necessarily need to reveal biological mechanisms behind the observed associations, possible mechanisms of the MRD dynamics in treatment response can be proposed based on our results. In particular, the MRD status appears to be influenced by the genetic variation of genes involved in pharmacokinetics/pharmacodynamics of anti-leukemic drugs (*RFC* and potentially *VDR*, acting as pleiotropic transcriptional regulator) as well as the genes involved in anti-tumor immunity of the host (*IL15* and potentially *VDR*).

MRD-associated polymorphisms, being accessible for the uncomplicated and cost-effective analysis at the time of diagnosis, and adding prognostic information to the existing prognostic factors, might potentially improve the risk assignment in pediatric ALL.

## Additional Information

**How to cite this article**: Dawidowska, M. *et al*. Association of germline genetic variants in *RFC, IL15* and *VDR* genes with minimal residual disease in pediatric B-cell precursor ALL. *Sci. Rep*. **6**, 29427; doi: 10.1038/srep29427 (2016).

## Figures and Tables

**Table 1:** Distribution of patients according to clinical features identified at diagnosis and early induction, presented in prognostic categories and resulting classification into risk groups.

Clinical features	Patients	
	N	%
	**174**	**100**
Sex		
Male	99	56.9
Female	75	43.1
Immunophenotype		
pro-B	14	8.0
common BCP-ALL	126	72.4
pre-B	33	19.0
B-lineage not otherwise specified	1	0.6
*Age at diagnosis* (*years*)		
<1 or >=6	89	51.1
1–5	85	48.9
*WBC at diagnosis* (*cells*/*ul*)		
>=50 000	26	14.9
<50 000	148	85.1
*Prednisone response at day 8*		
Poor	18	10.3
Good	156	89.7
*Bone marrow morphology at day 15*		
M3	9	5.2
M1/M2	165	94.8
*Bone marrow morphology at day 33*		
M2/M3	8	4.6
M1	166	95.4
*Fusion genes*		
*t*(*9*;*22*)/*BCR-ABL*		
Positive	5	2.9
Negative	164	94.3
Unknown	5	2.9
*t*(*4*;*11*)/*MLL-AF4*		
Positive	6	3.4
Negative	156	89.7
Unknown	12	6.9
*ALL IC-BFM 2002*/*2009 risk group*		
HR	37	21.3
IR	82	47.1
SR	55	31.6

WBC, white blood cell count; SR, standard risk; IR, intermediate risk; HR, high risk. Features in italics were used for the identification of MRD-associated clinical covariates in the study group.

**Table 2:** Distribution of patients according to MRD status at day 15, day 33 and week 12.

Time point	MRD monitoring method	Patients N	MRD status	
			Positive: >=10^−4^ N (%)	Negative: <10^−4^ N (%)
Day 15	FC	135	114 (84.4)	21 (15.6)
	FC/RQ	154	131 (85.1)	23 (14.9)
Day 33	FC	128	28 (21.9)	100 (78.1)
	FC/RQ	150	43 (28.7)	107 (71.3)
Week 12	FC	117	5 (4.3)	112 (95.7)
	FC/RQ	135	7 (5.2)	128 (94.8)

N, the number of patients with available MRD data obtained by flow cytometry (FC) and FC or real-time quantitative PCR (FC/RQ).

**Table 3:** Association of the clinical covariates with MRD status at different time points.

Time point	Clinical covariates	MRDFC			MRDFC/RQ		
		Negative	Positive	*P*	Negative	Positive	*P*
**Day 15**	ALL IC-BFM 2002/2009 risk group						
	SR	11	32	0.067	12	36	**0.044**^1^
	IR	9	60		9	64	
	HR	1	22		2	31	
**Day 33**	t(9;22)/*BCR-ABL* and/or t(4;11)/*MLL-AF4*						
	negativity	95	19	**0.001**^1^	100	27	**<0.001**^1^
	positivity	2	6		2	9	
	Bone marrow morphology at day 15						
	M1/M2	100	24	**0.002**^1^	106	35	**0.001**^1^
	M3	0	4		1	7	
	Prednisone response						
	good	95	25	>0.1	100	34	**0.017**^1^
	poor	5	3		7	9	
	Bone marrow morphology at day 33						
	M1	98	25	0.069	105	39	0.057
	M2/M3	2	3		2	4	
	ALL IC-BFM 2002/2009 risk group						
	SR	40	3	**<0.001**^1^	44	6	**<0.001**^1^
	IR	52	12		53	14	
	HR	8	13		10	23	
**Week 12**	t(9;22)/*BCR-ABL* and/or t(4;11)/*MLL-AF4*						
	negativity	97	2	**0.021**^1^	107	2	**<0.001**^1^
	positivity	5	2		5	4	
	Bone marrow morphology at day 15						
	M1/M2	107	2	**0.001**^1^	121	3	**0.007**^1^
	M3	0	2		2	2	
	ALL IC-BFM 2002/2009 risk group						
	SR	35	0	**<0.001**^1^	41	0	**<0.001**^1^
	IR	61	0		65	0	
	HR	11	4		17	6	

SR, standard risk; IR, intermediate risk; HR, high risk. Only P-values <0.1 were included in the table.

^1^Results statistically significant (P < 0.05, 2-sided Fisher exact probability test).

**Table 4:** Distribution of the genotypes among patients with MRD_FC_-positive and MRD_FC_-negative status.

*Gene*Polymorphism	Genotype	MRD_FC_ day 15			MRD_FC_ day 33			MRD_FC_ week 12		
		Neg. N (%)	Pos. N (%)	*P*	Neg. N (%)	Pos. N (%)	*P*	Neg. N (%)	Pos. N (%)	*P*
*MDR1* rs3789243	CC	4 (21.1)	39 (34.2)	0.522	33 (33.7)	7 (25.0)	0.395	35 (33.3)	1 (25.0)	0.595
	CT	10 (52.6)	49 (42.9)		45 (45.9)	12 (42.9)		47 (44.8)	2 (50.0)	
	TT	5 (26.3)	26 (22.8)		20 (20.4)	9 (32.1)		23 (21.9)	1 (25.0)	
*MDR1* rs2235046	AA	4 (20.0)	24 (21.1)	0.647	24 (24.2)	3 (10.7)	0.186	20 (18.87)	0 (0.0)	0.665
	AG	8 (40.0)	56 (49.1)		47 (47.5)	13 (46.4)		51 (48.1)	3 (75.0)	
	GG	8 (40.0)	34 (29.8)		28 (28.3)	12 (42.9)		35 (33.0)	1 (25.0)	
*MDR1* rs1045642	CC	4 (20.0)	24 (21.1)	0.421	20 (20.2)	8 (28.6)	0.634	26 (24.5)	0 (0.0)	0.677
	CT	11 (55.0)	46 (40.4)		41 (41.4)	10 (35.7)		44 (41.5)	2 (50.0)	
	TT	5 (25.0)	44 (38.6)		38 (38.4)	10 (35.7)		36 (33.9)	2 (50.0)	
*VDR* rs2228570	CC	6 (31.6)	35 (30.7)	0.904	32 (32.7)	9 (32.1)	0.660	31 (29.5)	1 (25.0)	0.811
	CT	10 (52.6)	56 (49.1)		45 (45.9)	15 (53.6)		51 (48.6)	3 (75.0)	
	TT	3 (15.8)	23 (20.2)		21 (21.4)	4 (14.3)		23 (21.9)	0 (0.0)	
*VDR* rs1544410	AA	0 (0.0)	20 (17.5)	0.078	13 (13.1)	7 (25.0)	0.303	15 (14.15)	1 (25.0)	0.064
	AG	9 (45.0)	53 (46.5)		48 (48.5)	11 (39.3)		55 (51.9)	0 (0.0)	
	GG	11 (55.0)	41 (35.9)		38 (38.4)	10 (35.7)		36 (33.9)	3 (75.0)	
*NR3C1* rs6198	AA	15 (75.0)	88 (77.2)	0.809	79 (79.8)	19 (67.8)	0.374	83 (78.3)	3 (75.0)	1.0
	AG	5 (25.0)	25 (21.9)		19 (19.2)	9 (32.1)		22 (20.8)	1 (25.0)	
	GG	0 (0.0)	1 (0.9)		1 (1.0)	0 (0.0)		1 (0.9)	0 (0.0)	
*NR3C1* rs41423247	CC	4 (21.1)	15 (13.2)	0.185	14 (14.3)	4 (14.3)	0.990	16 (15.2)	0 (0.0)	0.377
	CG	4 (21.1)	49 (42.9)		40 (40.8)	11 (39.3)		42 (40.0)	3 (75.0)	
	GG	11 (57.9)	50 (43.9)		44 (44.9)	13 (46.4)		47 (44.8)	1 (25.0)	
*GSTP1* rs1695	AA	8 (42.1)	34 (29.8)	0.563	35 (35.7)	4 (14.3)	0.089	31 (29.5)	1 (25.0)	0.827
	AG	6 (31.6)	45 (39.5)		34 (34.7)	14 (50.0)		41 (39.1)	1 (25.0)	
	GG	5 (26.3)	35 (30.7)		29 (29.6)	10 (35.7)		33 (31.4)	2 (50.0)	
*GSTM1* gene deletion	del/del	6 (31.6)	52 (45.6)	0.254	38 (38.8)	15 (53.6)	0.162	45 (42.9)	1 (25.0)	0.435
	del/wt, wt/wt	13 (68.4)	62 (54.4)		60 (61.2)	13 (46.4)		60 (57.1)	3 (75.0)	
*GSTT1* gene deletion	del/del	5 (26.3)	21 (18.4)	0.421	18 (18.4)	6 (21.4)	0.718	20 (19.1)	0 (0.0)	0.439
	del/wt,wt/wt	14 (73.7)	93 (81.6)		80 (81.6)	22 (78.6)		85 (80.9)	4 (100.0)	
*CCR5* rs333	del/del	0 (0.0)	1 (0.9)	0.303	1 (1.0)	0 (0.0)	1.0	1 (0.96)	0 (0.0)	0.999
	del/wt	1 (5.6)	23 (20.2)		18 (18.4)	5 (17.86)		21 (20.2)	1 (25.0)	
	wt/wt	17 (94.5)	90 (78.9)		79 (80.6)	23 (82.1)		82 (78.6)	3 (75.0)	
*TPMT* rs1142345	AA	19 (100.0)	106 (92.9)	0.651	91 (92.9)	27 (96.4)	1.0	100 (95.2)	4 (100.0)	1.0
	AG	0 (0.0)	7 (6.1)		6 (6.1)	1 (3.6)		4 (3.8)	0 (0.0)	
	GG	0 (0.0)	1 (0.9)		1 (1.0)	0 (0.0)		1 (0.9)	0 (0.0)	
*MTHFR* rs1801133	CC	9 (45.0)	52 (45.6)	0.909	44 (44.4)	13 (46.4)	0.647	47 (44.3)	2 (50.0)	1.0
	CT	9 (45.0)	47 (41.2)		43 (43.4)	10 (35.7)		42 (39.6)	2 (50.0)	
	TT	2 (10.0)	15 (13.2)		12 (12.1)	5 (17.9)		17 (16.0)	0 (0.0)	
*TYMS* rs3474033	2R/2R	4 (22.2)	25 (22.1)	0.946	19 (19.4)	10 (37.0)	0.138	25 (24.3)	1 (25.0)	0.835
	2R/3R	8 (44.4)	46 (40.7)		43 (43.9)	8 (29.6)		40 (38.8)	1 (25.0)	
	3R/3R	6 (33.3)	42 (37.2)		36 (36.7)	9 (33.3)		38 (36.9)	2 (50.0)	
*RFC* rs1051266	AA	4 (21.1)	26 (22.8)	0.622	19 (19.4)	11 (39.3)	0.086	25 (23.8)	4 (100.0)	0.004^1^^,^^2^
	AG	10 (52.6)	47 (41.2)		42 (42.9)	10 (35.7)		46 (43.8)	0 (0.0)	
	GG	5 (26.3)	41 (35.9)		37 (37.8)	7 (25.0)		34 (32.4)	0 (0.0)	
*IL15* rs10519613	AA	0 (0.0)	1 (0.9)	1.0	1 (1.0)	0 (0.0)	0.029^1^	0 (0.0)	0 (0.0)	1.0
	AC	4 (20.0)	27 (23.7)		17 (17.2)	11 (39.3)		25 (23.6)	1 (25.0)	
	CC	16 (80.0)	86 (75.4)		81 (81.8)	17 (60.7)		81 (76.4)	3 (75.0)	
*intergenic* rs3862227	AA	5 (27.8)	33 (28.9)	0.932	27 (27.6)	10 (35.7)	0.519	33 (31.7)	2 (50.0)	0.681
	AG	8 (44.4)	54 (47.4)		44 (44.1)	13 (46.4)		45 (43.3)	2 (50.0)	
	GG	5 (27.8)	27 (23.7)		27 (27.6)	5 (17.9)		26 (25.0)	0 (0.0)	
*NALCN* rs7992226	AA	12 (66.7)	51 (44.7)	0.207	47 (47.9)	14 (50.0)	0.670	50 (48.1)	3 (75.0)	0.799
	AG	5 (27.8)	47 (41.2)		38 (38.9)	12 (42.9)		39 (37.5)	1 (25.0)	
	GG	1 (5.6)	16 (14.0)		13 (13.3)	2 (7.1)		15 (14.4)	0 (0.0)	
*CCDC85C* rs11160533	CC	0 (0.0)	2 (1.8)	0.843	2 (2.0)	0 (0.0)	0.617	2 (1.9)	0 (0.0)	1.0
	CT	5 (27.8)	38 (33.3)		34 (34.7)	7 (25.0)		29 (27.9)	1 (25.0)	
	TT	13 (72.2)	74 (64.9)		62 (63.3)	21 (75.0)		73 (70.2)	3 (75.0)	
*intergenic* rs4888024	AA	9 (50.0)	50 (43.9)	0.865	46 (46.9)	9 (32.1)	0.373	46 (44.2)	1 (25.0)	0.453
	AG	7 (38.9)	52 (45.6)		42 (42.9)	15 (53.6)		47 (45.2)	2 (50.0)	
	GG	2 (11.1)	12 (10.5)		10 (10.2)	4 (14.3)		11 (10.6)	1 (25.0)	
*intergenic* rs9871556	CC	2 (11.8)	26 (24.1)	0.433	22 (23.7)	3 (11.1)	0.182	22 (22.0)	0 (0.0)	0.813
	CT	12 (70.6)	58 (53.7)		53 (56.9)	15 (55.6)		57 (57.0)	3 (75.0)	
	TT	3 (17.7)	24 (22.2)		18 (19.4)	9 (33.3)		21 (21.0)	1 (25.0)	

Neg. N, number of patients with MRD-negative results; Pos. N, number of patients with MRD-positive results.

^1^Results statistically significant at α = *P* < 0.05 (Pearson χ^2^ test or, if expected frequencies <5, Freeman-Halton extension of 2-sided Fisher’s exact probability test).

^2^Results significant after Benjamini-Hochberg procedure at false discovery rate, FDR = 0.1.

**Table 5:** Distribution of allele frequencies among patients with MRD_FC_-positive and MRD_FC_-negative status.

*Gene* Polymorphism	Allele	MRD day 15							MRD day 33							MRD week 12						
		MAF	Neg.	Pos.	OR	95% CI	*P*	Power	MAF	Neg.	Pos.	OR	95% CI	*P*	Power	MAF	Neg.	Pos.	OR	95% CI	*P*	Power
*MDR1* rs3789243	T	0.45	20	101	0.71	0.35–1.42	0.34	0.16	0.45	85	30	1.50	0.83–2.73	0.17	0.26	0.45	93	4	1.25	0.30–5.16	1.00	0.06
	C		18	127						111	26						117	4				
*MDR1* rs2235046	A	0.44	16	104	1.25	0.63–2.49	0.51	0.10	0.44	95	19	0.55	0.29–1.03	0.06	0.46	0.42	91	3	0.79	0.18–3.42	1.00	0.06
	G		24	124						103	37						121	5				
*MDR1* rs1045642	C	0.42	19	94	0.77	0.39–1.52	0.45	0.11	0.42	81	26	1.25	0.68–2.27	0.45	0.11	0.44	96	2	0.4	0.07–2.04	0.30	0.25
	T		21	134						117	30						116	6				
*VDR* rs2228570	T	0.44	16	102	1.11	0.55–2.23	0.76	0.06	0.44	87	23	0.87	0.47–1.59	0.66	0.07	0.45	97	3	0.69	0.16–3.00	0.72	0.08
	C		22	126						109	33						113	5				
*VDR* rs1544410	A	0.38	9	93	**2.37**	**1.07–5.21**	**0.03^1^**	0.70	0.38	74	25	1.35	0.74–2.46	0.32	0.16	0.39	85	2	0.49	0.09–2.52	0.48	0.17
	G		31	135						124	31						127	6				
*NR3C1* rs6198	G	0.12	5	27	0.94	0.33–2.60	1.00	0.05	0.12	21	9	1.61	0.69–3.75	0.26	0.15	0.11	24	1	1.11	0.13–9.49	1.00	0.05
	A		35	201						177	47						188	7				
*NR3C1* rs41423247	C	0.34	12	79	1.14	0.55–2.39	0.70	0.07	0.34	68	19	0.96	0.51–1.80	0.92	0.05	0.35	74	3	1.10	0.25–4.74	1.00	0.05
	G		26	149						128	37						136	5				
*GSTP1* rs1695	G	0.49	16	115	1.39	0.69–2.80	0.34	0.17	0.49	92	34	1.74	0.95–3.2	0.06	0.44	0.49	103	3	0.62	0.14–2.67	0.72	0.11
	A		22	113						104	22						107	5				
*CCR5* rs333	del	0.10	1	25	4.31	0.56–32.84	0.14	0.88	0.10	20	5	0.86	0.30–2.41	0.77	0.06	0.11	23	1	1.14	0.13–9.76	1.00	0.05
	wt		35	203						176	51						185	7				
*TPMT* rs1142345	G	0.03	0	9	N/A	N/A	0.36	N/A	0.03	8	1	0.42	0.05–3.49	0.68	0.22	0.03	6	0	N/A	N/A	1.00	N/A
	A		38	219						188	55						204	8				
*MTHFR* rs1801133	T	0.33	13	77	1.05	0.51–2.16	0.88	0.05	0.33	67	20	1.08	0.58–2.02	0.79	0.05	0.35	76	2	0.59	0.11–3.02	0.75	0.12
	C		27	151						131	36						136	6				
*TYMS* rs3474033	2R	0.43	16	96	0.92	0.45–1.87	0.82	0.06	0.43	81	28	1.52	0.83–2.79	0.16	0.28	0.43	90	3	0.77	0.18–3.32	1.00	0.07
	3R		20	130						115	26						116	5				
*RFC* rs1051266	A	0.43	18	99	0.85	0.42–1.69	0.64	0.08	0.43	80	32	**1.93**	**1.05–3.52**	**0.03^1^**	0.57	0.47	114	0	N/A	N/A	**<0.01**^**1,2,3**^	N/A
	G		20	129						116	24						96	8				
*IL15* rs10519613	A	0.12	4	29	1.31	0.43–3.95	0.79	0.08	0.12	19	11	**2.30**	**1.02–5.18**	**0.04**^**1**^	0.56	0.12	25	1	1.06	0.12–9.05	1.00	0.05
	C		36	199						179	45						187	7				
*intergenic* rs3862227	G	0.47	18	108	0.9	0.44–1.81	0.76	0.06	0.47	96	23	0.69	0.38–1.27	0.24	0.22	0.46	97	2	0.38	0.07–1.93	0.29	0.30
	A		18	120						96	33						111	6				
*NALCN* rs7992226	G	0.32	7	79	2.19	0.92–5.23	0.07	0.53	0.32	64	16	0.82	0.42–1.58	0.56	0.09	0.32	69	1	0.28	0.03–2.38	0.28	0.47
	A		29	149						132	40						139	7				
*CCDC85C* rs11160533	C	0.18	5	42	1.4	0.51–3.81	0.51	0.12	0.18	38	7	0.59	0.24–1.41	0.23	0.23	0.16	33	1	0.75	0.09–6.36	1.00	0.06
	T		31	186						158	49						175	7				
*intergenic* rs4888024	G	0.33	11	76	1.13	0.53–2.43	0.74	0.06	0.33	62	23	1.50	0.81–2.77	0.18	0.23	0.33	69	4	2.01	0.48–8.29	0.44	0.17
	A		25	152						134	33						139	4				
*intergenic* rs9871556	T	0.49	18	106	0.85	0.41–1.76	0.67	0.99	0.49	89	33	1.71	0.92–3.17	0.08	0.42	0.5	99	5	1.70	0.39–7.30	0.72	0.13
	C		16	110						97	21						101	3				

MAF, minor allele frequency; Neg., number of alleles in the group of patients with MRD-negative status; Pos., number of alleles in the group of patients with MRD-positive status; OR, odds ratio for the MRD-positive status for the minor allele; 95% CI, 95% confidence interval; Power, statistical power. ^1^Results statistically significant at α = 0.05 (Pearson χ^2^ test or, if expected frequencies <5, 2-sided Fisher’s exact probability test). ^2,3^Results significant after Benjamini-Hochberg procedure at false discovery rate, ^2^FDR = 0.1 and also at ^3^FDR = 0.05.

**Table 6:** Distribution of binary groups, representing allele positivity, in patients with MRD_FC_-positive and MRD_FC_-negative status.

Time point	Gene Polymorphism	Allele positivity	MRD_FC_ status		OR	95% CI	*P*		
			Negative	Positive					
Day 15	*VDR*	AA + AG	9	74	2.26	0.86–5.91	0.132		
		GG	11	40					
	rs1544410	GG + AG	20	94	N/A	N/A	0.080		
		AA	0	20					
Day 33	*IL15*	AA + AC	18	11	**2.91**	**1.17–7.27**	**0.024**^1,2^		
		CC	81	17					
	rs10519613	CC + AC	98	28	N/A	N/A	1.0		
		AA	1	0					
	*RFC*	AA + AG	61	21	1.82	0.71–4.69	0.264		
		GG	37	7					
	rs1051266	GG + AG	79	17	**0.37**	**0.15–0.92**	**0.043**^1,2^		
		AA	19	11					
Week 12	*RFC*	AA + AG	71	4	N/A	N/A	0.307		
		GG	34	0					
	rs1051266	GG + AG	80	0	N/A	N/A	**0.004**^1,2,3^		
		AA	25	4					

Negative/Positive, number of patients with MRD-negative/MRD-positive results, within each of the binary groups representing allele positivity; OR, odds ratio; 95% CI, 95% confidence interval. ^1^Results statistically significant at α = 0.05 (Pearson χ^2^ test or, if expected frequencies <5, 2-sided Fisher’s exact probability test). ^2,3^Results significant after Benjamini-Hochberg procedure at false discovery rate, ^2^FDR = 0.1 and also at ^3^FDR = 0.05.

**Table 7:** Additive effect of carrying risk alleles in *IL15* and *RFC* loci in association with MRD_FC_ positivity at day 33.

MRD by method			MRD day 33		OR	95% CI	*P*
	Number of Risk Alleles		Negative N (%)	Positive N (%)			
MRD_FC_	2 v. 1 vs. 0	2	8	8	N/A	N/A	**0.017**^1^
		1	63	16			
		0	27	4			
	1 vs. 0	1	63	16	1.71	0.52–5.61	0.425
		0	27	4			
	2 vs. 1	2	8	8	3.94	1.28–12.11	**0.024**^1^
		1	63	16			
	2 vs. 0	2	8	27	6.75	1.61–28.39	**0.012**^1^
		0	8	4			
MRD_FC/RQ_	2 v. 1 vs. 0	2	10	11	N/A	N/A	**0.031**^1^
		1	66	25			
		0	29	7			
	1 vs. 0	1	66	25	1.57	0.61–4.04	0.377
		0	29	7			
	2 vs. 1	2	10	11	2.90	1.09–7.68	**0.038**^1^
		1	66	25			
	2 vs. 0	2	10	11	4.56	1.39–14.97	**0.017**^1^
		0	29	7			

Negative/Positive, number of patients with MRD-negative/MRD-positive results, within each of the groups differing in the number of risk alleles; OR, odds ratio; 95% CI, 95% confidence interval; ^1^Results statistically significant (P < 0.05, 2-sided Fisher’s exact probability test or Freeman-Halton extension of 2-sided Fisher’s exact probability test, for 2 × 3 tables).

**Table 8:** Comparison of our study design with published reports on MRD-associated germline polymorphisms.

Study	Ref.	Therapeutic Protocol	MRD assessment		Patients	
			Method	Time Points	N	Ethnicity
Davies *et al*.	11	POD 9904	FC	day 8, day 28*	1197	Cauc., Hisp., Afr-Am., others
		POD 9905				
		POD 9906				
Stanulla *et al*.	12	ALL-BFM 2000	RQ	day 33*, day 78 (approx. week 12)	814	No Ethnic Data (German Trial)
Yang *et al*.	13	St Jude XIIIB	FC	day 19, day 46*	318	Cauc., Afr-Am., others
		COG P9906	FC	day 8, day 28*	169	
Present Study	—	ALL IC-BFM 2002	FC/RQ	day 15, day 33*, week 12	159	Cauc. of Slavic origin
		ALL IC-BFM 2009				

Ref., reference; N, number of patients studied; POD, Pediatric Oncology Group Protocols; ALL-BFM, Acute Lymphoblastic Leukemia Berlin-Frankfurt-Munster Study Protocol; St Jude XIIIB, St Jude Total Therapy XIIIB Protocol; COG, Children’s Oncology Group Trial Protocol; ALL IC-BFM; Acute Lymphoblastic Leukemia Intercontinental Berlin-Frankfurt-Munster Study Protocol; FC, flow cytometry; RQ, Real-Time Quantitative Polymerase Chain Reaction; Cauc., Caucasians; Hisp, Hispanics; Afr-Am, African Americans; * end of the remission induction treatment. A cut-off level of 10^−4^ was used in all the studies to define patients with MRD-positive and MRD-negative status.
